# Stress responses in high-fidelity simulation among anesthesiology students

**DOI:** 10.1038/s41598-021-96279-7

**Published:** 2021-08-23

**Authors:** Patryk Stecz, Marta Makara-Studzińska, Szymon Białka, Hanna Misiołek

**Affiliations:** 1 Department of Clinical Psychology and Psychopathology, Institute of Psychology, University of Łódź, Smugowa 10/12, 91-433 Łódź, Poland; 2 Department of Health Psychology, Faculty of Health Sciences, Jagiellonian University Collegium Medicum, Kopernika 25, 31-501 Kraków, Poland; 3 Department of Anaesthesiology, Intensive Care and Emergency Medicine, Faculty of Medical Sciences in Zabrze, Medical University of Silesia, Katowice, 3 Maja 13/15, 41-800 Zabrze, Poland

**Keywords:** Psychology, Neurophysiology, Risk factors, Endocrinology

## Abstract

Simulation sessions can produce high-fidelity emergency situations that facilitate the learning process. These sessions may also generate a complex stress response in the learners. This prospective observational study assessed psychological, physiological, immunological, and humoral levels of stress during high-fidelity simulation training. Fifty-six undergraduate medicine students who took part in a medical simulation session were assigned team roles (physician, nurse or assistant). Subsequently, each participant was assessed before the scenario (T_0_), after the procedure (T_1_), and two hours later (T_2_). Psychological stress and anxiety were measured at T_0_ and T_1_, using the State-Trait Anxiety Inventory (STAI) and Dundee Stress State Questionnaire (DSSQ). Cortisol, testosterone, secretory immunoglobulin class A (sIgA), alpha-amylase, and oxygen saturation level were measured at T_0_, T_1_, and T_2_, as was the physiological response indicated by heart rate (HR) and blood pressure (BP). It was found that the onset of task performance was related to increased anticipatory worry and higher oxygen saturation. The participants reported decreased worry, followed by increased emotional distress after the simulation training (T_1_). Participants trait anxiety predicted the intensity of worry, distress and task engagement. In contrast, no clear relationships were found between trait anxiety and biological stress markers. Testosterone levels were growing significantly in each phase of measurement, while physiological responses (BP, HR) increased at T_1_ and declined at T_2_. The levels of stress markers varied depending on the assigned roles; however, the trajectories of responses were similar among all team members. No evidence for prolonged cortisol response (T_1_, T_2_) was found based on psychological stress at the onset of simulation (T_0_). Regression analysis followed by receiver operating characteristics analyses showed uncertain evidence that initial state anxiety and worry predicted the levels of sIgA. Medical students are relatively resilient in terms of stress responses to medical simulation. The observed stress patterns and interrelationships between its psychological, physiological, hormonal, and immunological markers are discussed in accordance with theoretical concepts, previous research work, and further recommendations.

## Introduction

Clinical simulations are highly advanced active teaching methods that employ technology tools and produce high-fidelity scenarios, providing a beneficial learning environment in aviation or medicine. According to worldwide data, iatrogenic complications are the leading cause of death^1^, while simulation techniques enable medical students to learn how to manage emergency events under time pressure with high realism and no exposure to real risk at the same time. Furthermore, high-fidelity simulation (HFS) may effectively contribute to identifying and reducing the causes of human error in medical settings^2^. Although medical simulations are recognized as a highly cost-effective educational method, there is growing evidence showing that its participants are exposed to stress, which manifests in a complex way, including physiological response, humoral reactions, or psychological state of distress^3–5^.

Stress reaction, determined by stressor and person characteristics, is considered desirable for detecting an alarming signal and alleviating the response^6^. It may improve coping performance to maintain allostasis^7^; however, the too frequent experience of stress or ineffective coping is associated with possible alterations in neurotransmitter regulation and responsivity (the HPA axis, immunosuppression, etc.), increasing the vulnerability to further chronic disease^8,9^. According to transactional theory, psychological stress occurs when an individual appraises the situation and its demands as threatening or outweighing their own resources^10^. Psychological stress can be framed in three domains: cognitions (positive, neutral, or negative thoughts, worries), emotions (curiosity, excitement, fear, strain), and intentional responses (energy, active coping, fatigue, disengagement)^11^. Acute and prolonged physiological, humoral and immunological responses are considered to be inferential indicators of such stress. They could be measured by changes in the autonomic nervous system (heart rate variability), the rise of salivary cortisol^12^ or decreased production of the main antibody against pathogens (sIgA, secretory immunoglobulin class A)^13^. Recent research using noninvasive methods established another stress biomarker, α-amylase, which is a salivary digestive enzyme. In most cases, its level increases before and after acute psychological stress, which has been attributed to autonomic nervous system arousal^14,15^. According to the distinction introduced by Selye^16^, eustress (positive stress) and distress (negative stress) may lead to different outcomes of the humoral response. Distress associated with negative emotions increases the release of cortisol or norepinephrine (Brit *noradrenaline*)^17^. The cognitive activation theory of stress (CATS) also emphasizes the adaptive role of stress for coping processes, aiming to get a positive outcome of how an individual responds to a given stressor^7^. This theory explains sustained response to stress due to absent or ineffective coping, i.e., due to uncertainty or low sense of control. Together, these models bring a broader perspective for understanding the psychological basis of acute and prolonged neuroendocrine stress response^7,16,18^.

Assuming that stress reaction is rather complex in its manifestation, few studies addressed stress response changes related to HFS, emphasizing the equal importance of psychological, physiological and humoral components for stress assessment^19–22^. Hence, many questions regarding the dynamics and mechanisms of stress reaction to HFS remain to be addressed. Its variability could be attributed not only to individual factors (sex, previous experience, personality traits or highly individualized psychological appraisal) but also to their interplay with situational demands (i.e. assigned role^23^). For instance, a lead-physician role is associated with primary responsibility, while a nursing or assistant role requires less decision-making efforts but still develops other types of involvement (i.e., critical-thinking, communication).

The present study was focused on determining the dynamics of psychological, physiological, humoral, and immunological responses to HFS task performance and the relationships between them. We intended to control these patterns by including several relevant individual and situational factors, such as previous exposure to medical simulation or assigned team role. It is not known very well whether and how the assigned role affects the measurements of participants stress^24^. Our aims were also related to the inconsistencies in the literature regarding the patterns and length of stress response to HFS, which is adaptive, but may also lead to a wide range of short-term and long-term effects, possibly threatening the emotion regulation, psychological self and somatic health.

The following hypotheses were assumed for determining the dynamics of stress response and its interrelationships:

1. Physiological and cardiovascular system markers of stress, indicated by diastolic blood pressure, systolic blood pressure, mean blood pressure, heart rate per minute and oxygen saturation, vary at T_2_ (two hours after completing simulation task) compared to T_0_ (onset of the task) and T_1_ (as soon as the task is completed):
  1.1. Physiological (DBP, SBP, MBP, HRpm) stress will decrease at T_2_ compared to T_0_ and T_1_ while cardiovascular (SpO_2_) response will manifest by higher saturation at T_0_
2. Medical simulation is associated with humoral and immunological responses (differences between T_0_ and T_1_).
  2.1 Humoral response to medical simulation is lower at the onset of the task (T_0_) than at T_1_ (as soon as the task is completed).
  2.2. Immunological response to HFS indicated by IgA manifests by its lower level at T_1_ (when the task is completed) than at T_0_ (the onset of the task)
3. Prolonged humoral and immunological response (T_1_, T_2_) to HFS is predicted by psychological appraisal at the onset of the task (T_0_):
  3.1. Low task engagement (lack of motivation), negative cognitions (worrying) and negative emotional appraisal (distress and state anxiety) at the onset of the task (T_0_) predict humoral response indicated by cortisol, testosterone, α-amylase and IgA levels.

Regarding changes in psychological stress and anxiety between T_0_ and T_1_, the mean scores were compared with each other; however, no directional hypothesis was stated for several reasons. At the onset of a challenging situation, emotional distress and worry could be related to anticipation and primary appraisal, which contributes to perceiving a stressor as a threat^18^. Considering the individual differences, even when the task is finished and an external stressor disappears, according to Hobfoll^25^ conservation of resources theory, his or her stress may still escalate. It may occur due to their perceived loss of personal resources related to uncertain outcome or unsuccessful coping with situational demands. Considering the retrospective nature of internal stressors and principles derived from the conservation of resources hypothesis, we did not expect the overall strong decrement of psychological stress immediately after completing the task. The analyses were controlled for sex, previous experience with high-fidelity medical simulation and the assigned role.

## Materials and methods

The study was designed as a prospective, observational trial and was conducted at the Centre of Didactics and Medical Simulation of Medical University of Silesia, Katowice, Poland. It was approved by the University of Silesia Ethics Committee (approval No.: KNW/0022/KB1/35/1/17). All methods were performed in accordance with the relevant guidelines and regulations (including the Declaration of Helsinki). Raw data can be retrieved from Zenodo database (10. 5281/zenodo.4737778) or downloaded as part of the supplementary material.

### Study participants

In total, 56 medical faculty students (fifth and sixth year; 29 women; 27 men) scheduled to undergo high fidelity medical simulation as a part of standard academic program were enrolled in the study. Written informed consent was obtained from all participants prior to inclusion. The inclusion criterion was the willingness to participate. Exclusion criteria included known pregnancy, active infections, immune system diseases, metabolic or endocrine disturbances, and current use of any medication (except for oral contraceptives). Most participants had no or relatively modest previous medical simulation experience (mean number of hours 19.8 SD = 15.9).

### Methods

#### Procedure

At the beginning of scheduled classes in the simulation center, in the morning (9:00–12:00), students (four to six per simulation) were placed sitting at rest for 30 min. This procedure was used to eliminate the potential exposure to external stressors. According to differing responsibilities of medical team members, each team member was randomly assigned to different independent variable levels (one physician, two nurses and two assistants in each team). After sitting at rest, all participants were informed about their assigned roles and then measured for their stress levels (T_0_), including psychological variables. Since one occasion stress measurement offers rather low reliability, we assumed that the initial assessment at T_0_ would indicate the initial stage of anticipatory stress related to HFS and the assigned role. Simultaneously, data on sex, age, weight, associated diseases, stimulants used, and previous medical simulation experience were collected using a personal questionnaire. Before starting the scenario, participants were informed (for 10–15 min) by a physician instructor about the simulation room setup and manikin features.

Immediately after the end of the scenario (T_1_), approximately 40 min after T_0_, all stress levels were measured again, including psychological questionnaires. After the participants were placed sitting at rest for 120 min, their physiological, humoral, and immunological stress markers were measured again (T_2_). During sitting at rest, participants were briefly informed on the proper interventions without formal debriefing or giving information on individual performance. We allowed participants to exchange their opinions instead.

The simulated scenarios were performed using a high fidelity computer-based manikin simulator, with the possibility of remote control of vital signs (*SimMan 3G, SimBaby and SimJunior; Laerdal*). All medications and equipment required during the clinical scenarios were available. Standardized physiological responses to anticipated management steps were programmed and activated by a physician. When the simulation participants made an unexpected clinical decision, the physiologic response was entered manually by the monitoring physician. The scenario used was prepared and validated by experienced HFS instructors.

#### Scenario design

A 40-year-old man was transported to the emergency department. He is confused, with suspected carbon monoxide poisoning that has occurred at home (the whole family suffered). During the scenario, the patient’s condition worsens: he develops heart arrhythmia and has fluctuating levels of consciousness.

After five minutes, the next paramedical team brings in a four-year-old boy: heart rate (HR) 70/bpm, blood pressure (BP) 65/20 mmHg, with broad, stiff pupils and no response to peripheral pain stimulation. One minute after admission, the child suffers a cardiac arrest (asystole). During this time, the adult patient becomes nervous and aggressive.

After another five minutes, the third paramedical team arrives with an 11-month-old infant in cardiac arrest. The man is becoming more nervous and aggressive. He develops critical hypertension (BP 210/125 mmHg) with subsequent cardiac arrest (ventricular fibrillation (VF)).

Until the end of the scenario, the resuscitation of an adult man, four-year, and eleven-month-old child continues.

#### Data obtained and analyzed

At each of three-time points (T_0_, T_1,_ and T_2_), heart rate (HR; bpm), systolic blood pressure (SBP; mmHg), diastolic blood pressure (DBP; mmHg), mean blood pressure (MBP; mmHg), and Sp0_2_ (%) were measured. These parameters were assessed using a cardiomonitor (*Infinity Delta, Dräger; Germany*). At the same time, saliva was collected for immunoassay tests using the Salivette (*Sarstedt AG & Co, Germany*) system.

#### Obtaining material for biochemical assays

Saliva was collected from participants to perform laboratory tests, using a special disposable Salivette tube (*Sarstedt AG & Co, Germany*). Saliva was collected by placing a sterile tampon under the tongue or chewing for 30–45 s. The soaked saliva pad was then placed in a suspended insert with a perforated bottom. The insert with a tampon was placed in a centrifuge tube and closed with a stopper. Next, the tube was centrifuged (1000 × *g* for 10 min.) to obtain a ready to test saliva supernatant. Approximately 0.7 mL of the supernatant from every sample collected was used for further testing. Samples were frozen after centrifugation at − 85 °C until performing laboratory tests.

Saliva supernatant was tested for total protein levels, α-amylase activity, secretory immunoglobulin A (sIgA), cortisol, and testosterone levels.

#### Determination of α-amylase activity

Alpha-amylase activity assay was performed by a static method with *AMYLAZA* kit (*Aqua-Med Łódź, Poland*). The samples were diluted 100 times using 0.9% chloride solution. 2-chloro-4-nitrofenylo-maltotrioside is a substrate in this method. The reaction was performed in pH 6.0 MES buffer at 37 °C rendering a colored reaction product. The product was then analyzed spectrophotometrically at 405 nm. The results are presented in salivary α-amylase activity units (U/mL). Measurement imprecision of the method was 4.1%.

#### Determination of secretory immunoglobulin class A (sIgA) level

Determination of secretory immunoglobulin class A (sIgA) was performed using an ELISA (*Immunodiagnostic AG, Germany*) to determine the IgA levels. The analytical procedure was in accordance with the instructions provided by the manufacturer in the user manual attached to the kit. Absorbance readings were taken using a *µQuant* reader (BioTek, USA); the results were processed using the *KCJunior* program (*BioTek, USA*). The sensitivity of the method was 2.5 µg/ mL. The method's imprecision was 5.3%.

#### Determination of cortisol and testosterone levels

The commercial ELISA (*Diapra, Italy*) was used to determine cortisol and testosterone levels. The analytical procedure was in accordance with the manufacturer's instructions in the technical manuals supplied with the kits. Absorbance readings were taken using a *µQuant* reader (*Biotek, USA*), while the results were processed using *KCJunior* (*Biotek, USA*). The method’s sensitivity was 0.12 ng/mL for cortisol and 3.28 pg/mL for testosterone. The method's imprecision was 6.2% and 7.9%, respectively.

Total protein was determined using the Lowry method: This method uses the reactions between peptide bonds and tyrosine and Folin-Ciocalteu reagent. The absorbance of the resulting color was read at 650–750 nm, 30 min after the reagent addition. Bovine serum albumin water solution (*BSA – Sigma Aldrich, Germany*) at slightly basic pH was used as standard. The results obtained were presented in mg/mL. Measurement imprecision of the method was 6.5%.

### Psychological measurement tools

#### State-trait anxiety inventory (STAI)

For this study, STAI^26^, a self-report instrument, was used to measure state anxiety (related to task performance) and trait anxiety (dispositional vulnerability to experience anxiety). The participants are asked about the way they felt at the time of measurement and in general, respectively. The tool can be administered to assess different specific types of anxiety in a variety of contexts. The Cronbach’s α coefficient for the state anxiety scale varied from 0.83 to 0.92 and from 0.86 to 0.92 for trait anxiety, respectively, suggesting its high reliability.

#### Dundee stress state questionnaire (DSSQ)

The DSSQ was developed by Matthews et al.^27^ and provides a means for testing distinct elements of psychological stress in performance settings. Evidence has been found to confirm its three-factor structure (task engagement, worry, and distress) and adequate validity. Task engagement subscale is associated with positive affect and curiosity while worrying and distress have been related to negative affect, anger, and depression^11^. The questionnaire design was based on the trilogy of mind, emphasizing its three domains (affect—distress, worry—cognition, and task engagement—motivation). Task engagement can be hypothesized to play a constructive role as it appears to be influenced by primary stressor appraisal when the individual can classify whether the stressor is s challenge or a threat. High scores in DSSQ correspond with fatigue, distress, and worry, while low scores (apart from task engagement subscale indicating eustress) are related to success motivation, energetic arousal, success motivation, confidence, and peace of mind^11,27^.

### Statistical analyses

Statistica 13 software (StatSoft Inc., Tulsa, OK) and SPSS 25 were used in the statistical analysis. Homogeneity of variance was estimated using the Levene test. Sex differences, previous exposure to HFS and assigned roles were included in testing hypotheses. The significance level has been set at p < 0.05. To test hypotheses 1–2, we used repeated measures ANOVA, depending on the number of control variables interacting with hypothesized relationships. The testing for confounders revealed sex differences in SBP and Sp0_2_ levels, thus two-way (2 × 3) ANOVA was used to determine the sources of SBP and SpO_2_ variability and to test interactions. Hypothesis 3 was tested with correlation and multiple regression analysis, followed by ROC curves for predicting the relationship between initial psychological and prolonged immunological responses indicated by sIgA. By analogy with H_1_–H_2_, we used two-way ANOVA to determine the main effects of team roles and time conditions on psychological stress indicators. To study the intergroup differences, we used Tukey’s HSD post hoc pairwise comparison test. The Bonferroni correction was applied to compensate for multiple testing problem.

## Results

**Figure 1:**
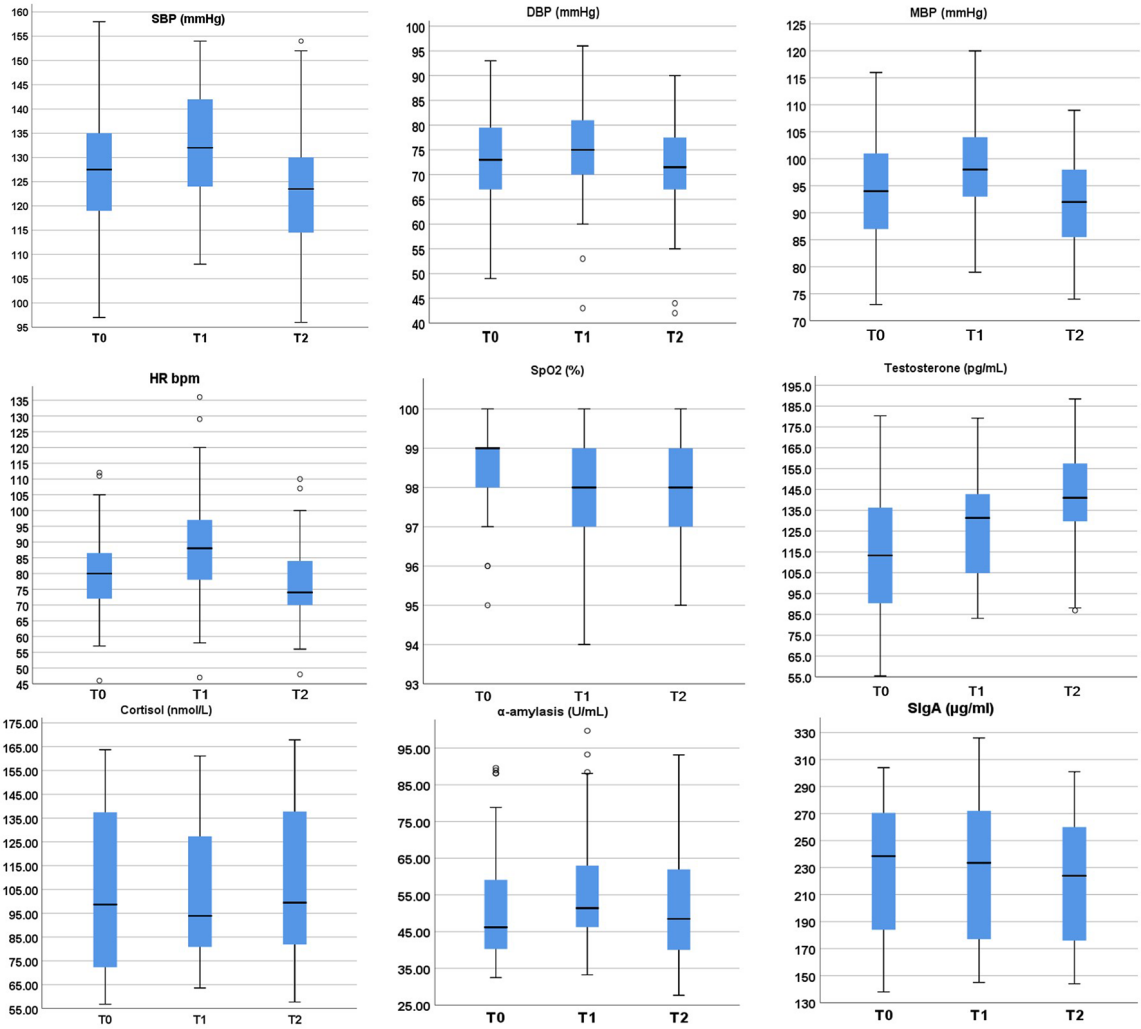
SBP, DBP, MBP, HR, SpO2, Testosterone, Cortisol, *α*-amylase and sIgA according to time levels. Description: Evolution of biological stress (blood pressure, heart rate, oxygen saturation, testosterone, cortisol, α-amylase and sIgA) from T_0_ to T_2_. The horizontal lines in each box are located at the sample 50th percentile. The top and bottom edges are located at the 75th and 25th percentiles. This original figure was produced by authors who performed statistical computations using IBM Corp. Released 2017. IBM SPSS Statistics for Windows, Version 25.0. Armonk, NY. IBM Corp. granted licenses to use SPSS statistical outputs by the corresponding author, representing the institution (University of Lodz).

Table 1 shows sociodemographic data and the results related to state anxiety, psychological, physiological, and humoral stress levels during different assessment phases. There was no significant difference in stress and anxiety levels between students from the fifth and sixth years. Percentile scores of biological stress markers for all time levels are presented in Fig. 1. In order to indicate possible sources of interindividual variability of the participants’ stress responses, we analyzed the effect of sex, previous exposure to medical simulation and assignment to team role before computing further analyses.

**Table 1:** Sample characteristics.

Time	Measure	Variable	% (N)	Mean	SD
		Females	51.8% (29)		
		Males	48.2% (27)		
		Ethnicity (white)	100% (56)		
		Age		25.00	2.70
	STAI-X2	Low trait anxiety (≤ 3 sten)^1^	26.8% (15)		
		Average trait anxiety (4–7 sten)	62.5% (35)		
		High trait anxiety (≥ 8 sten)	10.8% (6)		
		Trait anxiety (raw score)		39.30	8.63
T _0_	DSSQ	Task engagement		36.16	6.97
		Distress		31.61	6.79
		Worry		15.95	6.02
	STAI-X1	State anxiety		41.46	9.99
	Blood pressure	Systolic blood pressure		126.48	13.12
		Diastolic blood pressure		73.14	9.69
		Mean blood pressure		93.93	9.63
		Heart rate per minute		80.54	12.97
		SpO_2_		98.48	1.21
	Salivary measures	Cortisol		102.11	33.26
		Testosterone		111.51	32.23
		Alpha-Amylase		51.65	14.85
		SIgA		229.53	48.18
T _1_	DSSQ	Task engagement		40.84	5.21
		Distress		34.75	6.04
		Worry		8.29	5.63
	STAI-X1	State anxiety		40.23	9.58
	Blood pressure	Systolic blood pressure		131.95	11.38
		Diastolic blood pressure		75.80	9.69
		Mean blood pressure		98.41	9.26
		Heart rate per minute		88.88	17.42
		SpO_2_		97.89	1.44
	Salivary measures	Cortisol		104.57	28.24
		Testosterone		126.83	24.90
		Alpha-Amylase		56.29	14.95
		SIgA		227.16	50.33
T _2_	Blood pressure	Systolic blood pressure		122.61	11.26
		Diastolic blood pressure		71.57	9.61
		Mean blood pressure		91.71	8.54
		Heart rate per minute		76.46	12.39
		SpO_2_		98.20	1.38
	Salivary measures	Cortisol		106.82	31.51
		Testosterone		140.94	23.55
		Alpha-Amylase		51.81	15.57
		SIgA		220.14	46.85

*STAI-X1* State Trait Anxiety Inventory; *DSSQ* Dundee Stress State Questionnaire, *Sp0*_*2*_ peripheral oxygen saturation, *SIgA* Secretory Immunoglobulin A.

^1^Sten scores represent standardized results expressed from 1–10 with a mean of 5.5 and a SD of 2.

### Sex

We observed no significant effect of sex, except for state anxiety at T_0_, *t(54)* = *1.212, p* = *0.014, d* = *0.03*, indicating that woman (M = 42.83, SD = 7.24) scored slightly higher than men (M = 40.00, SD = 12.25). The effect size was too modest to be considered statistically reliable. T-tests revealed that the patterns of physiological stress were the same for both sexes. However, males reported higher SBP and lower SpO_2_ than females in each phase of observation (mean differences M_d_ at T_0_ of 9.30 mmHg and 1.00% SpO_2_, p = 0.007 and 0.001, respectively; M_d_ at T_1_ of 7.76 mmHg and 1.01% SpO_2_, p = 0.01 and 0.007, respectively; M_d_ at T_2_ of 10.63 mmHg and 1.02% SpO_2_, p = 0.0002 and 0.005, respectively).

### Previous exposure

Previous exposure to medical simulation was found to be correlated only with testosterone level at T_2_, *r(48)* = *− 0.37, p* = *0.011*.

### Team role condition

We identified significant differences in the levels of stress markers attributed to team role conditions; consequently, we analyzed main effects and interaction effects for these variables.

#### Hypothesis 1

Quantitative variables were presented as average values and SD (standard deviation values).

Two way repeated measures ANOVA was performed, with time condition as within-subjects factor and sex as between-subjects factor, to test the main effects and the interaction effect on SBP. The main effects were compared using the Bonferroni adjustment. The results showed a significant main effect of HFS phase (measurements taken at T_0_, T_1_ and T_2_) and sex on the SBP level (males reported higher scores). Pairwise comparisons revealed that the highest level of SBP was observed at T_1_ and that it varied significantly between T_0_ (p = 0.002) and T_2_ (p < 0.0001). The interaction effect was not statistically significant (Table 2).

**Table 2:** Main effects and interaction effects of time condition and sex on SBP level: two-way repeated measures ANOVA.

Effect	F	p	partial eta^2^	Mean sq	df	OP	Pairwise comparisons
Sex	14.659	.0003	.214	3571.142	1	.964	SBP_*M*_ > SPB_F_* p = .0003
Time^1^	19.378	< .0001	.264	1217.913	2	.999	SBP *T*_*0*_ <SBP *T*_1_* p = .002; SBP *T*_*1*_ > SBP *T*_2_* p = < .0001; SBP *T*_0_ > SBP *T*_2_ p = .057
Interaction^2^	0.460	.633	.008	28.889	2	.123	

* p < .05; *SBP* systolic blood pressure, *OP* observed power, *M* males, *F* females; ^1^Wilk’s Lambda = .541, F(2,53) = 22.445, p < .0001, η^2^ = .46; ^2^Sphericity assumption was not violated.

One-way repeated measures ANOVA showed the main effect for DBP changes (*Wilk’s Lambda* = *0.827, F(2,54)* = *5.653, p* = *0.002, η*^2^_*p*_ = *0.173*): the most significant was a decrease of DBP at T_2_ (pairwise comparisons: T_2_ vs T_1_ p = 0.005). Significant differences between time conditions were observed for MBP, a more complex indicator of blood pressure (*Wilk’s Lambda* = *0.593, F(2,54)* = *18.564, p* < *0.0001, η*^2^_*p*_ = *0.407*). MBP showed a significant increase from T_0_ to T_1_ (p = 0.0001) and a decrease from T_1_ to T_2_ (p < 0.0001).

There was a significant effect of time conditions on heart rate, *Wilk’s Lambda* = *0.475, F(2,54)* = *29.833, p* < *0.0001)*. Post hoc comparisons indicated that there was a significant increase of heartrate at T_1_ by 8.34 bpm (p < 0.0001) followed by its decrease of 12.41 bpm at T_2_ (p < 0.0001).

Two-factor ANOVA showed that the effects of time condition and sex on blood oxygen level were statistically significant, however the interaction effect was not significant (Table 3). Blood oxygen level significantly dropped at T_1_ (task completed); however, the effect was relatively modest. The first hypothesis was confirmed.

**Table 3:** Main effects and interaction effects of time condition and sex on SpO_2_ level: two-way repeated measures ANOVA.

Effect	F	p	partial eta^2^	Mean sq	df	OP	Sex	Pairwise comparisons	Time	
							F _vs_ M	T_0__vs_ T_1_	T_1__vs_ T_2_	T_2__vs_ T_0_
Sex	14.277	.0004	.209	42.916	1	.959	M < F* _p=.0004_			
Time^1^	5.646	.005	.095	4.859	2	.852		T_0_ > T_1_*	T_1_ < T_2_	T_0_ > T_2_
								^p=.005^	^p=.343^	^p= .227^
Interaction^2^	0.002	.998	.0001	0.002	2	.050				

* p < .05; *SpO*_*2*_ oxygen saturation; *OP* observed power; *M* males; *F* females; ^1^Wilk’s Lambda = .826, F(2,54) = 5.579, p < .006, η^2^ = .174; ^2^Sphericity assumption was not violated.

#### Hypothesis 2.

T-tests for independent groups found no evidence for sex differences in the analyzed measures. Table 4 illustrates the results of two-way repeated measures ANOVA with one between-subjects (team role) and one within-subjects factor (time). The humoral responses for three team role conditions were compared at the onset of simulation task (T_0_), immediately after the simulation (T_1_), and after two hours of sitting at rest (T_2_).

**Table 4:** Main effects and interaction effects of time condition and sex on humoral and immunological indicators: two-way repeated measures ANOVA.

Effect	F	p	Partial eta^2^	Mean sq	df	OP	Pairwise comparisons					
							Team role			Time		
		_vs_T_0_		Cortisol^1^			^nurse vs physician^	^assistant vs physician^	^assistant vs nurse^	T_o__vs_T_1_	T_1__vs_T_2_	T_2_
Team role	14.277*	.0004	.209	42.916	1	.959	^c^N > P	A > P*	A > N*			
							^p=.477^	^p<.0001^	^p<.0001^			
Time	2.276	.119	.041	352.964	1.635	.407						
Interaction ^a^	1.766	.155	.062	273.788	3.269	.466						
Testosterone^2^												
Team role	8.989	.0004	.253	10,221.572	2	.966	^c^N > P*	A > P	A < N*			
							^p=.002^	^p=.999^	^p=.003^			
Time ^b^	28.594	< .0001	.350	10,944.137	2	.999				T_0_ < T_1_*	T_1_ < T_2_*	T_0_ < T_2_*
										^p=.0003^	^p=.001^	^p< .0001^
Interaction	0.311	.870	.012	118.899	4	.117						
α-amylase^3^												
Team role	24.706*	< .0001	.482	8047.540	2	.999	^c^N > P *	A > P*	A < N*			
							^p<.0001^	^p=.444^	^p<.0001^			
Time ^b^	9.177*	.0002	.148	373.423	2	.973				T_0_ < T_1_*	T_1_ > T_2_*	T_0_ < T_2_
										^p=.001^	^p=.002^	^p= .999^
Interaction	0.514	.726	.019	20.908	4	.169						
sIgA^4^												
Team role	26.670	< .0001	.502	88,188.930	2	.999	^c^N > P*	A > P*	A < N*			
							^p<.0001^	^p=.002^	^p=.0002^			
Time ^b^	4.551	.013	.079	1394.435	2	.763				T_0_ > T_1_	T_1_ > T_2_	T_0_ > T_2_*
										^p=.626^	^p=.138^	^p=.031^
Interaction	1.072	.374	.039	328.379	4	.328						

* p < .05; OP- observed power; ^c^ Post hoc Tukey HSD; *N* nurse *P* physician, *A* assistant; ^a^ Sphericity assumption violated (Greenhouse–Geisser correction performed); ^b^ Sphericity assumed; ^1^Wilk’s Lambda = .943, F(2,52) = 1.571, p < .218, η^2^ = .057; ^2^Wilk’s Lambda = .541, F(2,52) = 22.028, p < .0001, η^2^ = .459; ^3^Wilk’s Lambda = .735, F(2,52) = 9.369, p = .0003, η^2^ = .265; ^4^Wilk’s Lambda = .873, F(2,52) = 3.797, p = .029, η^2^ = .127.

### Cortisol

A significant between-subjects effect was observed, indicating that cortisol levels were higher among assistants than in nurses and physicians. There was no main effect of time nor interaction effect (time x team role) (Table 4).

### Testosterone

**Figure 2:**
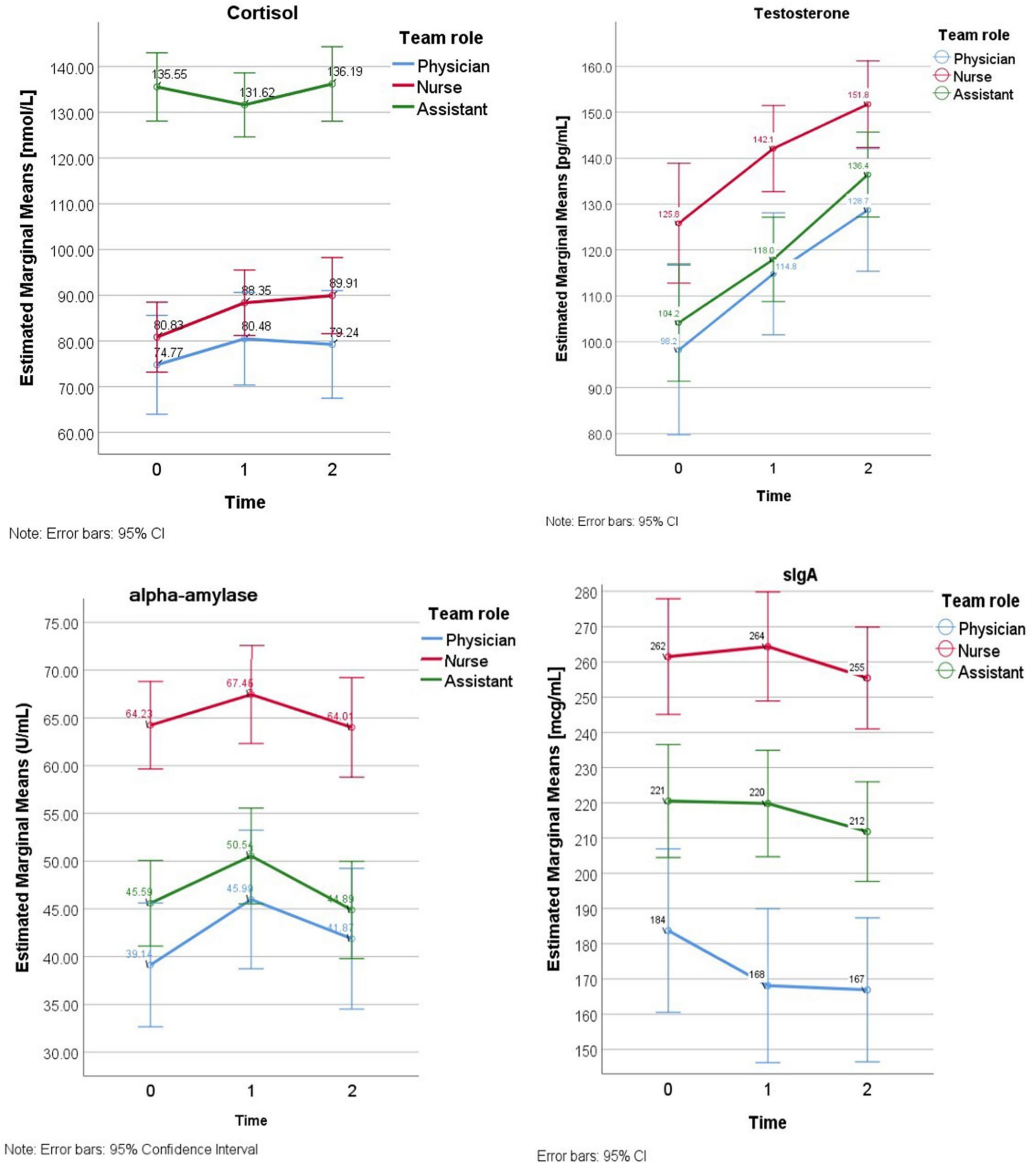
Estimated marginal means for cortisol, testosterone, *α*-amylase and sIgA according to assigned roles and time levels. Description: Mean differences (95% confidence intervals) in cortisol (nmol/L), testosterone (pg/mL), α-amylase (U/mL) and sIgA (mcg/mL) between participants assigned the role of a nurse, a physician or an assistant, controlled by time levels (T_0_, T_1_ and T_2_). This original figure was produced by authors who performed statistical computations using IBM Corp. Released 2017. IBM SPSS Statistics for Windows, Version 25.0. Armonk, NY. IBM Corp. granted licenses to use SPSS statistical outputs by the corresponding author, representing the institution (University of Lodz).

Two-way repeated-measures ANOVA revealed that the mean scores for testosterone varied across time levels and team roles. Further pairwise comparisons (with Bonferroni correction) showed that testosterone level increased significantly from T_0_ to T_1_ and from T_1_ to T_2_. The results of post hoc analyses suggested that testosterone levels were significantly higher among participants assigned the role of a nurse compared to participants with the role of an assistant or a physician (Fig. 2). The effect of interaction in two-way ANOVA was not observed.

### Alpha-amylase

The level of α-amylase was significantly higher among participants assigned the role of a nurse compared to those assigned physician or assistant tasks. The results showed significant main within-subjects effect (time) and no interaction effect (time x role), suggesting that the patterns of changes in α-amylase were similar in all participants: it increased from T_o_ to T_1_ and decreased at T_2_ (Table 4).

### sIgA

A significant between-subjects and within-subjects effects were observed. However, the observed power of time effect was low. Post hoc results indicated that sIgA levels were the highest among participants performing the nurse role. Significantly lower levels were reported for participants performing the role of an assistant. Those performing the role of a physician reported significantly lower sIgA than those assigned other roles; this was the only group reporting a decrease of sIgA from T_o_ to T_1_ (Fig. 2), but the interaction effect (time x team role) was not significant (Table 4).

Hypothesis 2 was partially confirmed.

#### Hypothesis 3.

To determine whether direct (T_0_/T_1_) and prolonged (T_2_) humoral responses to stress could be attributed to the psychological appraisal of stress (indicated by task engagement, worry, distress and anxiety), a multiple regression analysis was conducted to verify hypothesis 3. Before testing regression models, the assumptions were tested, including the linear relationship between independent and dependent variables (Table 5). The law of large numbers justified the use of the r-Pearson correlation coefficient.

**Table 5:** Pearson correlations between psychological indicators of stress and anxiety, and humoral arousal.

Variables	Time	Cortisol						Testosterone						sIgA level					
		T_0_		T_1_		T_2_		T_0_		T_1_		T_2_		T_0_		T_1_		T_2_	
		r	p	r	p	r	p	r	p	r	p	r	p	r	p	r	p	r	p
Trait anxiety		**− **.06	.68	.08	.58	.04	.80	.30*	.02	**.26**	.053	.13	.34	**− **.16	.23	**− **.14	.31	**− **.17	.21
Worry	T_0_	**− **.13	.35	**− **.06	.67	**− **.13	.34	.05	.70	**− **.03	.83	**− **.11	.42	**− **.28*	.04	**− **.14	.29	**− .26**	.051
Distress		.00	.99	.14	.29	.05	.71	.18	.18	.07	.59	.03	.83	**− **.17	.21	**− **.07	.61	**− **.16	.25
Task engagement		**− **.11	.43	**− **.07	.63	**− **.07	.61	**− **.15	.27	**− **.06	.66	.04	.75	.01	.96	.00	.99	.03	.83
State anxiety		.03	.85	.14	.30	.13	.32	.14	.29	.03	.84	.07	.59	**− **.31*	.02	**− **.30*	.03	**− **.29*	.03
Worry	T_1_			.07	.59	.08	.52			**− **.10	.49	**− **.07	.62			**− **.17	.20	**− **.12	.37
Distress				.22	.10	.24	**.08**			.11	.42	**− **.03	.81			**− **.14	.30	**− **.19	.16
Task engagement				**− **.14	.31	**− **.07	.61			**− **.18	.20	**− **.18	.19			**− **.03	.80	.06	.66
State anxiety				.15	.28	.16	.25			.21	.12	.13	.33			**− .23**	.09	**− .26**	.056

* p < .05; NA – not applicable; values in bold approximate to statistical significance; sIgA- secretory immunoglobulin class A.

While psychological indicators of stress and state anxiety did not correlate with humoral stress measures, trait anxiety predicted testosterone level at T_0_ and T_1_ (mild positive correlation). A few mild correlations were observed between psychological variables and IgA levels (Table 4). No associations were reported for the relationship between psychological responses and α-amylase activity. As there were no significant correlations for most variables, only one multiple regression model (enter method) was calculated for the variability of IgA at T_2_ based on the participants worry and state anxiety at T_0_. A significant regression equation was found (F(2,53) = 3.888, p = 0.027), with an R^2^ of 0.13. State anxiety was a significant predictor: the participant’s sIgA levels decreased by 5.262 µg/mL for each unit of state anxiety raw score (Standardized Beta = − 0.262). The association of worry and IgA was negative, but statistically insignificant.

**Figure 3:**
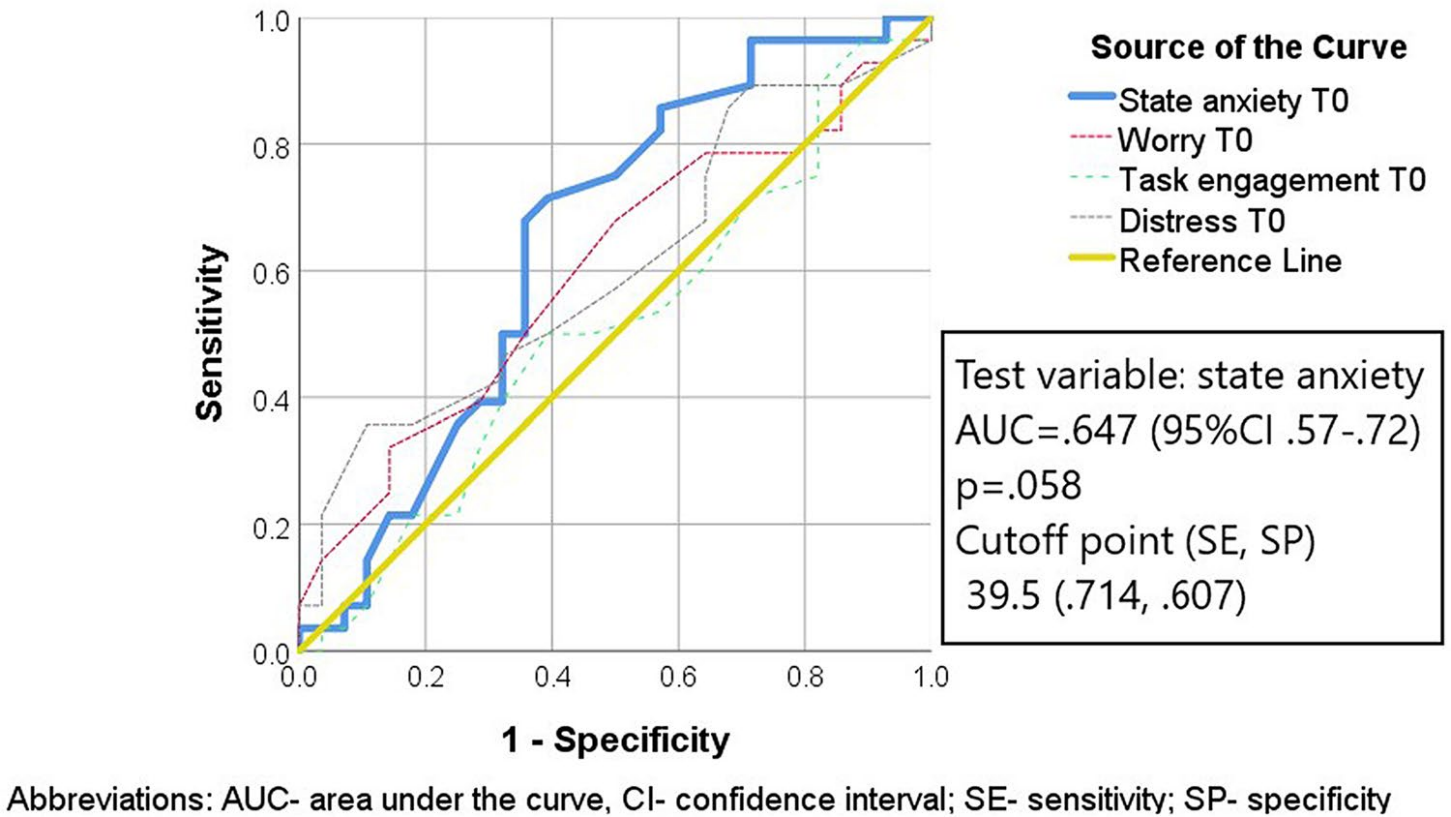
The receiver operating characteristics (ROC) curve for screening for sIgA at T_2_, using state anxiety and psychological indicators of stress at T_0_. Description: Receiver-operating characteristics displaying the ability of state anxiety, worry, distress and task engagement at T_0_ to predict secretory immunoglobulin class A (sIgA) level (high vs low) at T_1_. From this data, cutoff point can be generated to determine the greatest sensitivity and specificity for accuracy in terms of state anxiety raw score to predict decreased sIgA, indicating immunological stress response. This original figure was produced by authors who performed statistical computations using IBM Corp. Released 2017. IBM SPSS Statistics for Windows, Version 25.0. Armonk, NY. IBM Corp. granted licenses to use SPSS statistical outputs by the corresponding author, representing the institution (University of Lodz).

The diagnostic accuracy of state anxiety and other psychological stress indicators at T_0_ for predicting sIgA at T_2_ (a median split) was assessed with the area under the curve analysis of receiver operating characteristics curves (ROC). The cutoff points optimized for specificity and sensitivity, AUC, 95% confidence interval, and p-value obtained for state anxiety are reported in Fig. 3. The results showed that most variables did not report predictive power for sIgA (AUCs for worry, task engagement and distress were statistically insignificant and varied from 0.51 to 0.61). State anxiety was suggested to be marginally significant, approaching fair accuracy (AUC > 0.6).

The prolonged response to initial psychological stress/anxiety manifested by IgA levels only partially confirmed hypothesis 3. No evidence was found to support the hypothetical assumption regarding the predictive role of initial stress, worry, and anxiety induced by HFS for determining immediate or prolonged cortisol and adrenaline responses.

To assess the psychological impact of participating in HFS, the mean values of distress, worry, and state anxiety were compared before starting and after finishing the scenario, controlled for team role. Two factor ANOVA showed a significant decline in worry, an increase of distress and task engagement from T_0_ to T_1_ (Table 6). No significant change was observed between T_0_ and T_1_ conditions for state anxiety. Furthermore, Pearson correlation analyses confirmed that trait anxiety was associated with psychological stress and state anxiety at T_o_ (task engagement r = − 0.50, p = 0.0001, distress r = 0.74, p < 0.0001, worry r = 0.47, p = 0.0002, state anxiety r = 0.58, p < 0.0001) and at T_1_ (task engagement r = -0.31, p = 0.02, distress r = 0.48, p = 0.0001, worry r = 0.34, p = 0.011, state anxiety r = 0.60, p < 0.0001).

**Table 6:** Marginal means and standard errors for psychological distress, worry, task engagement and state anxiety in the interaction between time conditions and team roles.

Dependent variable	Time	Team role	M	SE	Dependent variable	Time	Team role	M	SE
Worry^1^	0^a^	Physician	17.64	1.83	Task engagement^3^	0^c^	Physician	38.09	2.12
		Nurse	15.23	1.29			Nurse	36.14	1.50
		Assistant	15.83	1.27			Assistant	35.26	1.46
	1	Physician	7.00	1.71		1	Physician	44.73	1.48
		Nurse	8.00	1.21			Nurse	39.86	1.05
		Assistant	9.17	1.18			Assistant	39.91	1.03
Distress^2^	0^b^	Physician	32.00	2.08	State anxiety^4^	0	Physician	42.64	3.04
		Nurse	31.18	1.47			Nurse	39.86	2.15
		Assistant	31.83	1.44			Assistant	42.44	2.10
	1	Physician	33.55	1.85		1	Physician	38.00	2.92
		Nurse	34.73	1.31			Nurse	40.27	2.07
		Assistant	35.35	1.28			Assistant	41.26	2.02

M- marginal mean, SE- standard error;

^1^Wilk’s λ = .428, F(1,53) = 70.974, p < .0001, η^2^ = .572, main effect of time (Greenhouse–Geisser correction): F(1) = 70.974, p < .0001, mean sq. = 1670.981, partial eta^2^ = .572, observed power = .999, no significant main effect of team role, no significant interaction effect;

^2^Wilk’s λ = .880, F(1,53) = 7.229, p < .010, η^2^ = .120, main effect of time (Greenhouse–Geisser correction): F(1) = 7.229, p < .010, mean sq. = 206.230, partial eta^2^ = .12, observed power = .752, no significant main effect of team role, no significant interaction effect; ^b^ pairwise comparison after applying Bonferroni correction: T_0_ < T_1_ p = .010;

^3^Wilk’s λ = .682, F(1,53) = 26.664, p < .0001, η^2^ = .318, main effect of time (Greenhouse–Geisser correction): F(1) = 7.26.664, p < .0001, mean sq. = 626.869, partial eta^2^ = .32, observed power = .998, marginally significant main effect of team role (p = .096), no significant interaction effect; ^c^ pairwise comparison after applying Bonferroni correction: T_0_ < T_1_ p < .0001;

^4^marginally significant model, Wilk’s λ = .880, F(1,53) = 7.229, p = .066, η^2^ = .062;

## Discussion

Task performance imposes high workload and personal concerns, which is why it may produce stress responses indicated by psychological, physiological, and humoral indices^11^. The objective of this study was to determine whether HFS is associated with stress responses as well as to find whether psychological appraisal at the onset of task performance (in terms of worries, negative emotions, and negative motivation) is associated with the level of cortisol, testosterone, α-amylase, and sIgA after completing the task. Our understanding on stress response complexity is that their nature could be both universal and interindividually variable, depending on personality traits or specific situational demands. The study participants undergone the same scenario setting, however their experiences depended on external factors (team role conditions) and individual factors (i.e. sex, differences in psychological appraisal of stress, previous experience with medical simulation). The results suggest that psychological stress indicators change over time while performing a task. Worrying manifested by self-focusing, negative thoughts, and decreased self-esteem was higher at the beginning of HFS. At the same time, emotional distress (which corresponds to high tension, unconfidence, and lack of positive emotions) turned out to escalate after completing the simulation. It shows that stress transaction during the observation could be characterized as turning from cognitive to emotional efforts in order to cope with psychological situational demands. The increase of emotional stress could be justified by the complexity and mutual influence of primary and secondary stress appraisal, which occurs before, during, and after the event^18,25^. The decline of worry and increase of task engagement could be attributed to the fact that the participants were not focused anymore on solving the problem during post-test (decrease of worry), but they could have been preoccupied with processing their performance (task engagement increment at T_2_), which is known to affect the stress response patterns^28^. Previous studies confirmed that distress and worry reactions during performance tasks change in opposite directions. In contrast, the drop of worry could be related to the shift from personal concerns to external stimuli or uncertainty reduction^11^.

The hypothesis regarding the physiological response to HFS was confirmed. The physiological arousal decreased two hours after sitting at rest, considering SBP, DBP, MBP, and HR. However, a decrease in oxygen saturation between T_0_ and T_1_ should be interpreted cautiously. The stress response is known to be associated with release of oxygen, which is a nutrient for muscular action. The arousal manifested by adrenaline secretion should be relatively short-term and followed by higher oxygen saturation^29^, which would take place at the onset of task performance. This pattern has been displayed as significantly higher SpO_2_ at T_0_. Oxygen maintenance at stress is multifaceted, with the vital role of the cardio-vascular system, sympathetic nervous system, glucocorticoids, or vasopressin^8,9^. Oxygen saturation is considered to be an ancillary method to detect stress, with a number of measurement approaches —oxygen saturation changes during psychological stress depending on specific areas of the body. Therefore, further studies might consider other techniques for comparison, such as measuring facial tissue oxygen saturation (StO_2_)^29^.

According to empirical findings, physiological and psychological responses to medical simulation may vary depending on scenario parameters. Harvey et al.^30^ found that the activation of the autonomic nervous system could be related to the problem difficulty level.

It was hypothesized that humoral response to task performance would be lower at the onset of HFS than at task completion. Engagement in HFS was associated with elevated testosterone level. In response to stress, testosterone may play an important role in developing control behavior, self-confidence, or aggression^31^ and in modulating social behavior^32^. It was previously reported that anxiety and stress can lead to decreased salivary testosterone level^33^. However, according to instability hypothesis by Zilioli et al.^34^, an increase of testosterone levels during task performance might be due to struggling to compete to improve the individual’s status, especially when it is uncertain. This hypothesis corresponds with our observations, considering the positive relationship between trait anxiety and testosterone during the early observation phases.

Regarding individual differences and their impact on interindividual variability in responding to stress, we found somewhat unexpected results for the role of trait anxiety. Trait anxiety, reflecting cross-situational vulnerability to stress, has been confirmed to explain the intensity of distress, worry and eustress (task engagement) related to HFS. However, almost no relationships were found between trait anxiety and other (physiological, cardiovascular system, humoral and immunological) stress markers. It suggests that personality related vulnerability to stress could have affected psychological responses rather than biological^3^. This inconsistency should be interpreted with caution, as our sample was non-clinical with almost 90% of participants displaying low to average vulnerability to stress and anxiety. Our findings suggest that handling students with increased levels of trait anxiety in a medical simulation could be focused mainly on managing their sense of fatigue, sources of worries and decline of motivation, i.e. by means of on-site debriefing or subsequent psychological counseling. On the other hand, participating in HFS seems to prevent high trait anxiety students from being exposed too quickly to *in-vivo* emergency settings which are more demanding in terms of stress.

Previous medical simulation experience was expected to interfere with participants’ stress reactions, however we found no evidence for this assumption. Bringing our findings in line with previous studies, physiological stress responses (increased heart rate and blood pressure) could be related to workload, regardless of past HFS experience. This is contrary to traditional tutorial-based training which is not associated with increased physiological reactions^35^. Homogeneous psychological responses among students with and without previous HFS experience may be explained by the fact that they worked together and gained practical skills during previous years of study. Also, it should be noted that psychological stress stemming from medical simulation might affect to a greater extent those who participate in simulation for the first time^36^. Exposure to novelty is considered as a significant source of emotional strain^3^, thus producing a non-linear effect of training on psychological stress.

In contrast with previous experience, role assignment could have affected biological stress responses to HFS. Statistically significant effects of role assignment on cortisol, testosterone, alpha-amylase and sIgA levels should be approached with some caution, considering no interaction effects and the absence of main effect of role assignment on psychological stress parameters. A possible explanation for role assignment effect might be that nurse tasks required high involvement (demands) and allowed lower control, yielding higher levels of humoral stress^37^. According to our study design and its findings, it is suggested that the optimal learning conditions, possibly affecting performance and knowledge related to realistic situations, could apply the exchange of task roles. At the end of the study, our participants shared positive feedback on being assigned different roles and were willing to replace each other in the future. Significant differences in humoral stress responses between team role conditions support the argument for role switching (i.e. lead physician—nurse), which could balance the exposure to stressful conditions across medical students.

The third hypothesis was only partially confirmed. It should be noted that the explanatory power of the proposed regression model is too low for precise predictions and that the relationships between psychological stress indicators were in most cases unrelated to cortisol, adrenaline, and α-amylase levels measured at different observation stages of. It has been established that lymphocyte production and activity (responsible for IgA secretion) may be shortly elevated when stressor occurs, and then inhibited due to glucocorticoid levels (a component of the secondary response to stress)^8,9^. Hence, the variability in sIgA secretion could be attributed to psychological factors, which have been reviewed in literature through the lens of the paradigm of immunological stress markers^38^. Several studies confirmed the negative relationship between stress and sIgA secretion^39,40^, which suggests that stress and anxiety may contribute to higher vulnerability of the immune defense system. This pathway by which anxiety could increase susceptibility cannot be extrapolated to long term effects on vulnerability to infectious disease as (a) sIgA levels may have increased after the observation, and (b) state anxiety at T_0_ may have been affected by many extensive factors outside the context of medical simulation. In general, the evidence from this study indicates that sIgA secretion may be considered as a weak immune marker of stress.

Cortisol level appeared to be unaffected by HFS phase and unrelated to psychological markers of stress, except for the main effect of the assigned role. According to the findings of Bauer and colleagues^3^, stress involves several types of emotional and body responses, which may be disassociated. As mentioned in the literature, cortisol has been shown to raise approximately five minutes following plasma cortisol increase, reaching its highest level more than half an hour after the onset of stressful event^41^. The initial measurement of salivary cortisol before the simulation might be, therefore, regarded as the most reliable indicator of baseline or anticipatory stress. Consequently, after the simulation, the second measurement would be associated with the HPA axis response intensity during the simulation. The third measurement could be interpreted as prolonged HPA response to a stressor, representing a potentially negative factor associated with cumulative or chronic stress^42^. Short term activity of stress hormones could be regarded as an optimal response as it may improve cognitive functions^3^. Previous works showing similar results (no changes in cortisol levels) explain this phenomenon, i.e., by increased pre-scenario cortisol level (related to participants’ expectancies), relatively low psychological demands of the scenario itself^30^ or efficient coping strategies utilized by the participants, preventing them from developing prolonged humoral responses, according to CATS^7^. In further research, it seems reasonable to determine the influence of the latter factor on humoral markers of stress by comparing subjective psychological demands of different HFS scenarios, including the present one, using independent expert assessment.

## Limitations

Our study, despite its experimental design and the advantages of using repeated measures, comes along with certain drawbacks linked to an incomplete control of the variables. First, due to the lack of baseline stress measurement, our results do not provide evidence for a causal link between the assigned role and participants’ stress. An important limitation lies in the fact that the initial changes in stress levels across team roles could be attributed to individual differences. However, we controlled this by splitting participants into multiple teams. Furthermore, we assumed that the initial measurement of stress before role assignment would be contaminated by confounding factors (*e.g.*, anticipatory stress related to HFS).

Greater time intervals in pretesting or post-testing stress markers would allow more accurate assessment of their baseline levels. The results suggest, on the one hand, that psychological stress appraisal before task performance has limited explanatory power for humoral stress responses observed during and shortly after HFS. Considering the small sample size and relatively few repeated measures, which are regarded as study limitations, the analyses were not sensitive to modest relationships; thus not all the results have reached statistical significance level. Finally, the study participants were fifth and sixth-year medicine students, which should be highlighted in generalizing the present results.

## Conclusion

The results of this investigation show that psychological stress trajectory in response to HFS could be characterized by an increase of distress, a decline of worry, and an increase of task engagement. Testosterone elevation was also observed for three-time levels (onset of simulation, at completion, two hours later). Stress responses of students exposed to HFS were predicted by situational factors (assigned role), and individual determinants (trait anxiety and sex), while no evidence was detected for previous HFS experience effect. Further research work needs to be done to explain this mechanism. State anxiety and worrying partially explained the variability of sIgA levels, suggesting that psychological stress symptoms are related to immunological responses. Psychological and biological stress reactions were rather inconsistent: anticipatory psychological distress did not predict prolonged cortisol response to exposure to HFS. Our research revealed that medical students were generally resistant to acute stress; however, the best practice should involve better management of students’ wellbeing, i.e., emotional distress reduction after HFS lessons or switching the assigned roles.

## Supplementary Information

Supplementary Information.
